# Carcinoid Tumors of Digestive Organs: a Clinicopathologic Study of 13 Cases

**DOI:** 10.4021/gr2009.01.1268

**Published:** 2009-01-20

**Authors:** Tadashi Terada

**Affiliations:** Department of Pathology, Shizuoka City Shimizu Hospital, Miyakami 1231 Shimizu-Ku, Shizuoka 424-8636, Japan. Email: piyo0111jp@yahoo.co.jp

**Keywords:** Digestive organs, Carcinoid, Clinicopathology, Immunohistochemistry

## Abstract

The author investigated clinicopathologic features of 13 cases of carcinoid tumor in the digestive organs. The 13 cases of carcinoid were identified from 18,267 pathological specimens of digestive organs in the last 10 years in our pathology laboratory. The tumor locations were rectum in 9 cases, duodenum in 2 cases, liver in 1 case, and stomach in 1 case. The age of the patients ranged from 52 to 82 years with a mean of 63 years. Male to female ratio was 7 : 6. The presenting symptoms were abnormal pain in 3 cases and asymptomatic in 10 cases. None of the cases showed carcinoid syndrome. The diameter ranged from 5 mm to 25 mm in gastrointestinal carcinoids, and 60 mm in the hepatic carcinoid. The treatment was endoscopic mucosal resection in 10 cases and surgical resection in 3 cases. The outcome is good except for hepatic atypical carcinoid which showed metastases and died of systemic metastasis. Histologically, 12 carcinoid tumors were typical carcinoids, and one (liver) was atypical carcinoid. Organoid pattern was present in 12 cases. Trabecular arrangement, ribbon arrangement, rosette formation, and pseudoglandular arrangement were recognized in 12 cases, in 8 cases, in 7 cases, and in 5 cases, respectively. Immunohistochemically, tumor cells were positive for at least one of pan-neuroendocrine markers including chromogranin, synaptophysin, neuron-specific enolase, CD56, and glucagon. Of these, synaptophysin was positive in 11/13 (85%), neuron-specific enolase 10/13 (80%), chromogranin 8/13 (62%), CD56 6/13 (46%), and glucagon 4/13 (31%). In summary, the author reported the incidence of digestive organ carcinoid tumors, and the clinicopathologic features of the 13 cases with carcinoid.

## Introduction

Carcinoid tumors, also called neuroendocrine tumors (NET), are relatively rare in the digestive organs [1-8]. The incidence is reported to be less that 0.1% of all digestive organ tumors [1-3]. Carcinod tumors are potentially malignant tumor, but the malignant potential depends on tumor size and morphologies [3]. In general, carcinoid tumors less than 20 mm are benign, and those more than 20 mm have malignant potential [3]. The author herein reports a clinicopathology of 13 cases of carcinoid tumors obtained from 18,267 archival pathologic specimens of digestive organs.

## Materials and Methods

The author retrospectively reviewed 18,267 pathological specimens of digestive organs pathologic specimens in the last 10 years in our pathology laboratory in search for carcinoid tumors. In carcinoid tumors, clinical and pathologic records were reviewed, and the pathologic slides were re-examined.

An immunohistochemical study was performed using Dako Envision methods (Dako Corp. Glostrup, Denmark), as previously described [9, 10]. The antibododies used were anti-cytokeratin (AE1/3, Dako), anti-cytokeratin (polyclonal wide, Dako), carcinoembrionic antigen (polyclonal, Dako), chromorgranin (DAK-A3, Dako), synaptophysin (polyclonal, Dako), neuron-specific enolase (BBS/NC/VI-H14, Dako), CD56 (MOC-1, Dako), and glucagon (polyclonal, Dako).

## Results

A total of 13 carcinoid tumors (0.07%) were found among the 18,267 digestive organ’s pathologic specimens in the last 10 years in our pathology laboratory.

The carcinoid tumor locations were rectum in 9 cases, duodenum in 2 cases, liver in 1 case, and stomach in 1 case. The age of the patients ranged from 52 to 82 years with a mean of 63 years. Male to female ratio was 7 : 6. The presenting symptoms were abnormal pain in 3 cases and asymptomatic in 10 cases. None of the cases showed carcinoid syndrome. The diameter ranged from 5 mm to 25 mm in gastrointestinal carcinoids, and 60 mm in the hepatic carcinoid. The treatment was endoscopic mucosal resection in 10 cases and surgical resection in 3 cases. The outcome is good except for hepatic atypical carcinoid which showed metastases and died of systemic metastasis.

**Figure 1:**
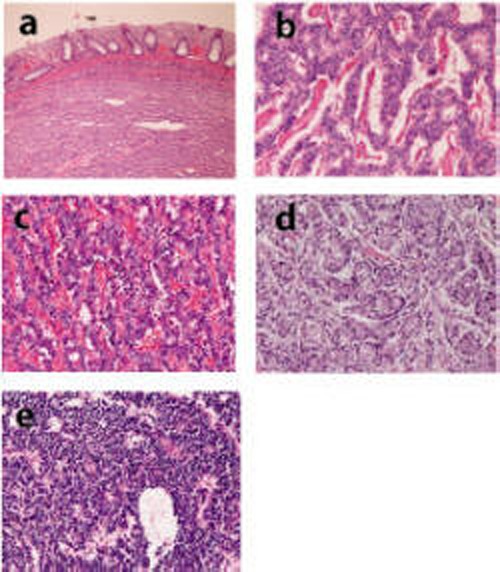
(a) Carcinoid is located in the submucosa. HE, x 40. (b) Carcinoid of the colon. HE, x 200. (c) Carcinoid of the duodenum. HE, x 200. (d) Carcinoid of the stomach. HE, x 200. (e) Atypical carcinoid or neuroendcrine carcinoma of the liver. HE, x 200.

**Figure 2:**
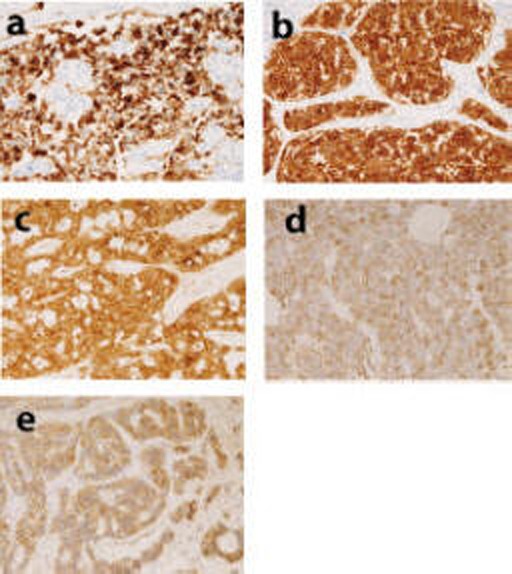
(a) Expression of chromogranin in carcinoid. Immunostaining, x 200. (b) Expression of synaptophysin in carcinoid. Immunostaining, x 200.(c) Expression of neuron-specific enolase in carcinoid. Immunostaining, x 200. (d) Expression of CD56 in carcinoid. Immunostaining, x 200. (e) Expression of glucagon in carcinoid. Immunostaining, x200.

Histologically, the carcinoids were located in the submucosa (Figure 1A), and were grossly identified as submucosal tumor. Of the 13 carcinoids, 12 carcinoid tumors were typical carcinoids (Figures 1B, 1C and 1D). The liver carcinoid was an atypical carcinoid composed of atypical endocrine cells (Figure 1E). Organoid pattern was present in 12 cases. Trabecular arrangement, ribbon arrangement, rosette formation, and pseudoglandular arrangement were recognized in 12 cases, in 8 cases, in 7 cases, and in 5 cases, respectively. Immunohistochemically, tumor cells were positive for cytokeratin in 7 cases and negative for carcinoembryonic antigen in all cases. The tumor cells were positive for at least one of pan-neuroendocrine markers including chromogranin (Figure 2A), synaptophysin (Figure 2B), neuron-specific enolase (Figure 2C), CD56 (Figure 2D), and glucagon (Figure 2E). Of these, synaptophysin was positive in 11/13 (85%), neuron-specific enolase 10/13 (80%), chromogranin 8/13 (62%), CD56 6/13 (46%), and glucagon 4/13 (31%).

## Discussion

In the present series, only 13 carcinoids were found in the 18,267 pathologic specimens in the last 10 years in our pathology laboratory. The incidence was 0.07%. The incidence of carcinoids of digestive organs is reported to be less than 0.1% of all digestive organ tumors [1-3]. Therefore, the incidence of 0.07 % in the present series is compatible with other institutes [1-3]. These findings suggest that carcinoids of digestive organs are rare.

In the present series, the locations of carcinoids were rectum in 9 cases, duodenum in 2 cases, liver in 1 case, and stomach in 1 case. A study of larger series indicated that the frequency of carcinoids of digestive organs is highest in the colon, followed in order by ileum, rectum, appendix, stomach, duodenum, jejunum, pancreas, and liver. Carcinoid of the liver is very rare [11]. The locations of carcinoids in this study were somewhat different from other institutes.

In the present series, the age of the patients ranged from 52 to 82 years with a mean of 63 years. Male to female ratio was 7 : 6. These findings are compatible with other institutes (1-8).

The presenting symptoms were abnormal pain in 3 cases and asymptomatic in 10 cases in the present series. None of the cases showed carcinoid syndrome. In general, patients with carcinoids present with non-specific symptoms such as abdominal pain and nausea [3]. The presenting symptoms depend on locations and size of carcinoids [3]. The tumor size of the present study was small. Therefore, asymptomatic patients predominate in the present study.

The prognosis of the present series is good except for the hepatic atypical carcinoid. In other institutes, five-year survival of carcinoids depends on tumor stage and tumor biologic behavior; it ranges from 60% to 98% [3].

In the present series, the treatment was endoscopic mucosal resection in 10 cases and surgical resection in 3 cases. It seems that the best choice of treatment is endoscopic resection in small carcinoids. In larger carcinoids, surgical operation may be effective.

Histopathologically, all cases but the hepatic atypical carcinoid showed typical features of carcinoids, such as trabecular, ribbon, rosette, and pseudoglandular arrangements, in the present series. The liver carcinoid of the present series may be a neuroendocrine carcinoma rather than atypical carcinoid.

Immunohistochemically, tumor cells were positive for cytokeratin in 7 cases and negative for carcinoembryonic antigen in all cases in the present series, suggesting that carcinoid tumor cells may be negative for cytokeratin. In the present series, the tumor cells were positive for at least one of pan-neuroendocrine markers including chromogran, synaptophysin, neuron-specific enolase, CD56, and glucagon. Of these, synaptophysin was positive in 11/13 (85%), neuron specific enolase 10/13 (80%), chromogranin 8/13 (62%), CD56 6/13 (46%), and glucagon 4/13 (31%). These findings suggest that these pan-neuroendocrine markers are useful diagnostic tools in the carcinoid diagnosis.
